# Supplementary material for: Between belief and fear - Reinterpreting prone burials during the Middle Ages and early modern period in German-speaking Europe
Source: PLoS One. 2020 Aug 31;15(8):e0238439. doi: 10.1371/journal.pone.0238439 (PMC7458347; doi:10.1371/journal.pone.0238439)
Supplement: S1 File — (DOCX) [file pone.0238439.s001.docx]

**Supporting Information S1 Reference list to Table 1**

in order of the occurrence in Table 1

1. Grossschmidt K, Rendl B, Staub A, Urban M. Skelette, Mumien und Scheintod. In: Sauer F, al. e, editors. Die Pfarrkirche von Altlichtenwarth. Horn: Ferdinand Berger und Söhne; 2014. p. 92-9.

2. Sauer F, al. e. Die Pfarrkirche von Altlichtenwarth. Horn: Ferdinand Berger und Söhne; 2014.

3. Weber J. Untersuchungen auf dem Kirchhof der Marienkirche in Anklam. Archäologische Berichte aus Mecklenburg-Vorpommern. 1999;6:135-48.

4. Steintor Mi. Anklam – Siedlung am Fluss. Eine über 1000-jährige Geschichte im Spiegel der Archäologie. Anklam2009.

5. Wintergerst M. Stadtkirche Bayreuth : Beobachtungen zu Baugeschichte und Bestattungen in Spätmittelalter und früher Neuzeit. Das Archäologische Jahr in Bayern. 2013;2012:145-7.

6. Heinrich E. Die Dorfkirche in Tempelhof. Eine baugeschichtliche Untersuchung. Der Bär von Berlin Jahrbuch des Vereins für die Geschichte Berlins. 1954;4:45-88.

7. Burkhardt A. Ein Neben(be)fund löst das Rätsel um historische Insulaner. Aus der anthropologischen Begutachtung der Skelette vom Borkumer "Walfängerfriedhof". Archäologie in Niedersachsen. 2017;20:105-8.

8. Burkhardt A. Ungewöhnliche Befunde an Skeletten von der Nordseeinsel Borkum - Eine anthropologische Spurensuche mit überraschendem Ergebnis Mitteilungen der Berliner Gesellschaft für Anthropologie, Ethnologie und Urgeschichte. 2017;38:53-72.

9. Bader C, Langenegger E. Ein Bestattungsareal in Bülach vom Mittelalter bis in die Neuzeit. In: Gisler J, editor. Archäologie im Kanton Zürich. 2. Zürich und Egg: Baudirektion Kanton Zürich Hochbauamt; 2013. p. 7-36.

10. Eggenberger P, Ulrich-Bochsler S, Utz Tremp K, Pahud de Mortanges E, Kühn M, Schlumbaum A, et al. Das mittelalterliche Marienheiligtum von Oberbüren. Archäologische Untersuchungen in Büren an der Aare, Chilchmatt. Bern: Archäologischer Dienst des Kantons Bern; 2019.

11. Jungklaus B. Sit tibi terra levis - "Die Erde möge Dir leicht sein". Sonderbestattungen auf dem Friedhof des mittelalterlichen Diepensee, Lkr. Dahme-Spreewald. In: Biermann F, Müller U, Terberger T, editors. „Die Dinge beobachten “ Archäologische und historische Forschungen zur frühen Geschichte Mittel- und Nordeuropas Festschrift für Günter Mangelsdorf zum 60 Geburtstag. Rahden/Westf.: Verlag Marie Leidorf; 2008. p. 379-87.

12. Jungklaus B. Sonderbestattungen vom 10.-15. Jh. in Brandenburg aus anthropologischer Sicht. Ethnographisch-Archäologische Zeitschrift. 2009;50:197-214.

13. Bohnet S, Seidel A. Spätmittelalter-/frühneuzeitlicher Friedhof inmitten früheisenzeitlicher Siedlungsbefunde in Echenbrunn. Das Archäologische Jahr in Bayern. 2018;2017:138-9.

14. Binding G. Die Ausgrabungen 1964/65. In: Binding G, Janssen W, Jungklaass FK, editors. Burg und Stift Elten am Niederrhein Archäologische Untersuchungen der Jahre 1964/65. Rheinische Ausgrabungen. Düsseldorf: Rheinland-Verlag; 1970. p. 1-234.

15. Jungklaass FK. Die anthropologische Bearbeitung der Skelettfunde südlich des Rundbaues. In: Binding G, Janssen W, Jungklaass FK, editors. Burg und Stift Elten am Niederrhein. Rheinische Ausgrabungen. Düsseldorf: Rheinland-Verlag; 1970. p. 297-306.

16. Maier RA. Neuzeitliche Zigeuner-Bestattungen und Pferdeopfer-Deponierungen bei der Stadt Erding in Oberbayern. Jahresbericht der Bayerischen Bodendenkmalpflege. 1980;21:229-41.

17. Maier RA, Winghart S. Nichtchristliches Totenbrauchtum auf einem neuzeitlichen Bestattungsplatz bei der Stadt Erding, Oberbayern. Das Archäologische Jahr in Bayern. 1981;1981:196-7.

18. Maier RA. Nochmals zum nichtchristlichen Totenbrauchtum auf einem neuzeitlichen Bestattungsplatz bei der Stadt Erding. Das Archäologische Jahr in Bayern. 1988;1988:168-71.

19. Fehring GP, Scholkmann B. Die Stadtkirche St. Dionysius in Esslingen a.N. Stuttgart: Konrad Theiss Verlag; 1995.

20. Meier T. Mithras im Mittelalter? Ein außerordentlicher Fund des 2./3. und 13. Jahrhunderts vom Petersberg. Das Archäologische Jahr in Bayern. 2002;2001:146-8.

21. Meier T. Die Konstruktion des Wissens: Eine römische Gemme aus einem mittelalterlichen Grab vom Petersberg bei Flintsbach/Inn, Oberbayern. In: Keupp J, Schmitz-Esser R, editors. Neue Alte Sachlichkeit, Studienbuch Materialität des Mittelalters Ostfildern: Jan Thorbecke Verlag; 2015. p. 335-65.

22. Mohr RH, Meier T, Wiechmann I, Grupe G. Morphologische und molekulargenetische Untersuchung einer ungewöhnlichen Dreifachbestattung am Petersberg/Kleinen Madron bei Flintsbach a. Inn, Lkr. Rosenheim. Bericht der Bayerischen Bodendenkmalpflege. 2001; 1998/99(39/40):319-29.

23. Lösch S. Paläopathologisch-anthropologische und molekulare Untersuchungen an mittelalterlichen und frühneuzeitlichen Bevölkerungsgruppen. Ernährung und Gesundheitszustand süd- und nordbayerischer Bevölkerungsstichproben [Dissertation]. München: Ludwig-Maximilians-Universität 2009.

24. Jenisch B, Bohnet S. Knochenarbeit im Schatten des Münsters. Zur Ausgrabung auf dem mittelalterlichen Friedhof Freiburgs. Denkmalpflege in Baden-Württemberg. 2015;1:39-44.

25. Wintergerst M. Ausgrabungen 2013 Alt-Füssen Veröffentlichungen des Historischen Vereins Alt-Füssen 2015; 2013:5-18.

26. Schulze R. Grabow, Lkr. Ludwigslust-Parchim, Fpl. 32. Bodendenkmalpflege in Mecklenburg-Vorpommern. 2015; 63:366-7.

27. Kaute P. Bemerkenswerte Bestattungen vom Kirchhhof des Klosters Eldena, Hansestadt Greifswald. Archäologische Berichte aus Mecklenburg-Vorpommern. 2011;18:149-61.

28. Kaute P. Bemerkenswerte Bestattungen auf dem Friedhof des ehemaligen Zisterzienserklosters Eldena. Greifswalder Beiträge zur Stadtgeschichte, Denkmalpflege, Stadtsanierung. 2011 (2012);5:44-5.

29. Ansorge J. Ein Rosenkranz und andere Merkwürdigkeiten vom Friedhof der Jacobikirche in Greifswald Archäologische Berichte aus Mecklenburg-Vorpommern. 2003;10:180-94.

30. Jüngling P. Hanau-Kesselstadt. Zur Archäologie einer Pfarrkirche in Hanau: Hanauer Geschichtsverein 1844 e.V. ; 2004.

31. Nösler D. Untote und Bann. Zwei mittelalterliche Wiedergängerbestattungen aus dem Kreuzgang des Benediktinerklosters Harsefeld. Geschichte und Gegenwart. 2014;2014:11-20.

32. Selent A. Die hoch- bis spätmittelalterliche Wüstung Klein Hoym, Salzlandkreis, im Rahmen der B6n-Grabungen. Archäologie in Sachsen-Anhalt. 2018;9:297-319.

33. Berszin C. Der Spitalfriedhof Heiliggeist-Hospital in Konstanz. Vorläufiger Bericht über die Ausgrabungen 1995-1996. In: Brather S, Bücker C, Hoeper M, editors. Archäologie als Sozialgeschichte Studien zu Siedlung, Wirtschaft und Gesellschaft im frühgeschichtlichen Mitteleuropa Festschrift für Heiko Steuer zum 60 Geburtstag. Rahden/Westf.: Verlag Marie Leidorf GmbH; 1999. p. 127-34.

34. Berszin C. Kloster, Dorf und Vorstadt Petershausen. Anthropologische Untersuchungen. In: Röber R, editor. Kloster, Dorf und Vorstadt Petershausen: archäologische, historische und anthropologische Untersuchungen. Forschungen und Berichte der Archäologie des Mittelalters in Baden-Württemberg. Stuttgart2009. p. 117-90.

35. Guichon R, Thorimbert S, Bohny J. Lausanne VD, Vidy, Comité International Olympique. Jahrbuch Archäologie Schweiz. 2017;100:235-6.

36. Häckel M. Opfer - Hexe - Ausgestossene? Möglichkeiten der anthropologischen Datenerfassung mit FileMaker^TM^ am Beispiel einer Sonderbestattung aus Luppa, Landkreis Nordsachsen. Beiträge zur Archäozoologie und Prähistorischen Anthropologie. 2009;VII:183-94.

37. Häckel M. Rätselhafte Skelette - die Toten von Luppa und Belgern-Klosterhof. Ausgrabungen in Sachen. 2012;3:165-71.

38. Schneider M, Holze-Thier C, Thier B. Die Ausgrabungen auf dem Domherrenfriedhof von 1987 bis 1989. Die Stiftskirche "Alter Dom" und die Bestattungen im Dombereich. Teil 2: Die Bestattungen auf dem Domherrenfriedhof und im Johanneschor. Mainz: Philipp von Zabern; 2011.

39. Thier B. "Sonderbestattungen" im Mittelalter und in der Neuzeit - Christliche Diskriminierung als Phänomen "unehrlicher" Begräbnisse und die Grenzen der archäologischen Interpretation. In: Brink-Kloke H, Mielke DP, editors. Vom Umgang mit dem Tod Archäologie und Geschichte der Sepulkralkultur zwischen Lippe und Ruhr Beiträge zur Tagung im LWL-Museum für Archäologie Herne am 7 November 2014. Büchenbach 2018. p. 34–51.

40. Winkler S. Der Friedhof unter dem Parkplatz. In: Winkler S, editor. Die Stadt Münster: Ausgrabungen an der Stubengasse (1997-1999). Denkmalpflege und Forschung in Westfalen. Mainz: Verlag Philipp von Zabern; 2008. p. 139-52.

41. Hotz G. Die Bestattungsplätze des Klosters St. Johann : anthropologische Auswertung einer frühmittelalterlichen bis neuzeitlichen Skelettserie unter spezieller Berücksichtigung spurenelementanalytischer Fragestellungen [Dissertation]. Basel: Universität Basel; 2002.

42. Hensch M. Sankt Johans Freidhof in Nabburg – Gewöhnliche und ungewöhnliche Einblicke in die spätmittelalterliche Begräbniskultur Ostbayerns. In: Husty L, Irlinger W, Pechtl J, editors.,, …und es hat doch was gebracht!“ Festschrift für Karl Schmotz zum 65 Geburtstag. Internationale Archäologie - Studia honoraria. Rahden/Westf.: Marie Leidorf Verlag; 2014. p. 423-40.

43. Prehn B. Totenkrone und Eselsbegräbnis - Bestattungen und Bestattungsplätze in Spätmittelalter und Früher Neuzeit. In: Jöns H, Lüth F, Schäfer H, editors. Archäologie unter dem Strassenpflaster. 15 Jahre Stadtkernarchäologie in Mecklenburg-Vorpommern. Beiträge zru Ur- und Frühgeschichte Mecklenburg-Vorpommerns. Schwerin: Archäologisches Landesmuseum und Landesamt für Bodendenkmalpflege Mecklenburg-Vorpommern; 2005. p. 459-64.

44. Ernst B. Ausgrabungen und Baubefunduntersuchungen in der ehemaligen Kirchenburg Neukirchen b. Hl. Blut, Lkr. Cham. Bilanz der Jahre 1989 und 1990. In: Schmotz K, editor. Vortäge des 10 Niederbayerischen Archäologentages. Buch am Erlbach 1992. p. 133-65.

45. Schütte S. Die Grabkapelle des Otto von Northeim. In: Lüdtke H, Lüth F, Laux F, editors. Archäologsicher Befund und historische Deutung Festschrift für Wolfgang Hübener zu seinem 65. Geburtstag am 15 Juni 1989. Hammaburg. Neumünster: Wachholtz; 1989. p. 247-63.

46. Ungerath O. Gemeindefriedhof. Bestattungen und Siedlungsbefunde im Zentrum von Prenzlau, Kreis Uckermark. Archäologie in Berlin und Brandenburg. 2003;2002:128-33.

47. Alterauge A. Die Bestattungen im Bereich des Langhauses der Kirche des Klosters Elisabethenzell. wwwspessartprojektde [Internet]. 2014.

48. Bujard J. La Fille-Dieu à Romont, dossier archéologique d'un monstère de moniales cisterciennes. Cahiers d'Archéologie Fribourgeoise. 2018;20:140-213.

49. Wiedmann B. Friedhöfe, Seuchenopfer und Anatomieleichen - Aktuelle anthropologische Forschungen zu Mittelalter und früher Neuzeit in Westfalen. In: Otten T, Hellenkemper H, Kunow J, Rind M, editors. Fundgeschichten - Archäologie in Nordrhein-Westfalen. Schriften zur Bodendenkmalpflege in Nordrhein-Westfalen. Köln: Philipp von Zabern; 2010. p. 342-5

50. Alterauge A, Baeriswyl A, Blaser C, Brechbühl-Trijasse S, Lösch S, Plamondon M, et al. Eine ungewöhnliche Bestattung im Friedhof von Schüpfen. Archäologie Bern/Archéologie bernoise. 2017;2017:246-66.

51. Meyer S, Doswald S. Ehemaliger Friedhof bei der Pfarrkirche St. Matthias in Steinhausen: ausgewählte Resultate der anthropologischen Auswertung. Tugium: Jahrbuch des Staatsarchivs des Kantons Zug, des Amtes für Denkmalpflege und Archäologie, des Kantonalen Museums für Urgeschichte Zug und der Burg Zug. 2012;28:139-51.

52. Wittkopp B. Kloster und Friedhof der Dominikaner zu Strausberg. Mitteilungen der Berliner Gesellschaft für Anthropologie, Ethnologie und Urgeschichte. 2008;29:99-115.

53. Wittkopp B. Der Dominikanerfriedhof in Strausberg. Sonderbestattungen, Sicheln und ihre Interpretation. In: Beilke-Voigt I, Biermann F, editors. Glaube-Aberglaube-Tod Vom Umgang mit dem Tod von der Frühgeschichte bis zur Neuzeit. Ethnographisch-Archäologische Zeitschrift. Berlin2009. p. 179-96.

54. Stadler H. Die Heilerin vom Strader Wald. Eine Sonderbestattung des 17. Jahrhunderts aus Tarrenz in Tirol (Vorbericht). In: Kreissl E, editor. Kulturtechnik Aberglaube: Zwischen Aufklärung und Spiritualität Strategien zur Rationalisierung des Zufalls. Bielefeld: transcript; 2013. p. 359-92.

55. Jungklaus B. Der mittelalterliche bis frühneuzeitliche Friedhof der St.-Maria-Magdalenen-Kirche, Templin. Templiner Heimatkalender. 2007;2007:100-2.

56. Lutz D. Archäologische Beiträge zur Geschichte Ubstadts. In: Hildebrandt LH, editor. Archäologie und Wüstungsforschung im Kraichgau. Heimatverein Kraichgau Sonderveröffentlichung. Ubstadt-Weiher: Heimat 1997. p. 113-28.

57. Eggenberger P, Ulrich-Bochsler S. Unterseen: die reformierte Pfarrkirche: die Ergebnisse der archäologischen Forschungen von 1985 (mit Ergänzungen von 1998 und 2000). Bern: Paul Haupt AG; 2001.

58. Auberson L, Descoeudres G, Keck G, Stöckli W. La chapelle des Martyrs à Vérolliez. Vallesia. 1997;52:355-434.

59. Arnold S. Dorfsterben...: Vöhingen und was davon blieb. Archäologie eines mittelalterlichen Dorfes bei Schwieberdingen. Stuttgart: Gesellschaft für Vor- und Frühgeschichte in Württemberg und Hohenzollern; 1998.

60. Bulla A, Dubbi F-J, Grundmann B, Wiedmann B, Lissner B. Die vorstädtisceh Siedlung Hüffert auf dem Gelände des St. Petri-Hospitals in Warburg. Archäologie in Westphalen-Lippe. 2013;2012:138-42.

61. Prohaska R. Lebendig begraben – Ein Skelettfund aus dem Chor der Minoritenkirche in Wien: Opfer der Medizin, Übeltäter oder Wiedergänger? In: Kühtreiber K, Kühtreiber T, editors. Beiträge zur historischen Archäologie Festschrift für Sabine Felgenhauer-Schmiedt. Beiträge zur Mittelalterarchäologie Österreichs. Wien2003. p. 167–71.

62. Biermann F, Blum O, Hergheligiu C. Neue Forschungen zum Prämonstratenserstift Grobe auf der Insel Usedom - Ausgrabungen am Wilhelmshofer Priesterkamp im Jahre 2010. Bodendenkmalpflege in Mecklenburg-Vorpommern. 2015 [2017];63:53-?

63. Jungklaus B. Die Bestattungen beim spätmittelalterlichen Prämonstratenserstift Grobe (Wilhelmshof) auf der Insel Usedom – Ergebnisse der anthropologischen Analysen. Bodendenkmalpflege in Mecklenburg-Vorpommern 2017 [2015];63.

64. Jäggi C, Meier H-R, Windler R, Illi M. Die Stadtkirche St. Laurentius in Winterthur. Ergebnisse der archäologischen und historischen Forschungen. Zürich; Egg: Fotorotar AG; 1993.

65. Grünewald M. Pilgerzeichen, Rosenkränze, Wallfahrtsmedaillen. Die Beigaben aus Gräbern des 17. bis 19. Jahrhunderts aus dem Pfarrfriedhof bei St. Paul in Worms. Die Sammlung gotischer Pilgerzeichen im Museum der Stadt Worms. Worms: Stadtarchiv Worms; 2001.

66. Moser D, Seiler R, Bertschi M, Ohnsorg P, Langenegger E, Boeni T, et al. A deviant, prone burial from Fraumünster, Zürich (early modern period) and the evolution of rheumatoid arthritis. 1st Evolutionary Medicine Conference; University of Zurich 2015.
