# Supplementary material for: Between belief and fear - Reinterpreting prone burials during the Middle Ages and early modern period in German-speaking Europe
Source: PLoS One. 2020 Aug 31;15(8):e0238439. doi: 10.1371/journal.pone.0238439 (PMC7458347; doi:10.1371/journal.pone.0238439)
Supplement: Attachment — Submitted filename: Response to Reviewers.docx [file pone.0238439.s002.docx]

**Response to Reviewers**

First of all, we would like to thank the two reviewers and the editor for their valuable comments and constructive criticism. The manuscript underwent a language check by a native speaker.

We have integrated the followings comments in the manuscript.

1. Style requirements, tables and supporting information

Style of the title page and the main body were adapted to PLOS ONE’s style requirements. Tables were inserted in the manuscript and information on the Supporting information files was added.

2. Additional information on specimens

The study deals with archaeological specimens, of which the majority is published. We clarified that the specimens are stored in the respective heritage institutions of the states or cantons. In Table 1, we added the states/cantons to the countries. The statement „All necessary permits were obtained for the study, which complied with all relevant regulations“ was added to the material section.

3. Map (Figure 1)

Permission for publication was obtained from the copyright holder of Figure 1 (map). The copyright holder has added information on the map creation: Basic vector map of Europe, the Isohypses are produced by using Copernicus data and information funded by the European Union - EU-DEM layers. The bodies of water are based on data from [www.naturalearthdata.com](http://www.naturalearthdata.com) . Publication of the basic vector map at Zenodo: <http://doi.org/10.5281/zenodo.3457998>

4. Age distribution (reviewer #1)

A sentence on the possible bias of the age distribution was added to the discussion.

5. Geographical bias (reviewer #1)

Sentences have been added on the geographically imbalanced state of research.

6. Volkskunde (reviewer #1)

Indeed, Wiedergänger and Nachzehre are terms of German folklore. We have added expressions to make clear that we summarize the perceptions reported in the Handwörterbuch des deutschen Aberglaubens. In the reference list, we now indicate the original publication date of the book.

7. Medieval England (reviewer#2)

Two sentences have been added to the research history on deviant burials on the situation in Britain, and Gilchrist and Sloane 2005 have been cited.

8. Chronology (reviewer#2)

As requested, we have added centuries to chronological designations, where necessary.

9. Map (reviewer#2)

As requested, we have added a map of Europe to figure 1, indicating our study area.

We are looking forward to your decision and the timely publication of our manuscript.

Yours sincerely,

Amelie Alterauge & Sandra Lösch
